# Supplementary material for: Brain-to-gut trafficking of alpha-synuclein by CD11c+ cells in a mouse model of Parkinson’s disease
Source: Nat Commun. 2023 Nov 20;14:7529. doi: 10.1038/s41467-023-43224-z (PMC10658151; doi:10.1038/s41467-023-43224-z)
Supplement: Supplementary file 1 — Supplementary Information [file 41467_2023_43224_MOESM1_ESM.pdf]

Supplementary Information

Supplementary Figure 1: αSyn aggregation is specific to the ileum.

Supplementary Figure 2: αSyn localizes to the region of the ileum enriched with immune cells.

Supplementary Figure 3: $CD11c^+$ cells express classical markers for macrophages, dendritic cells, and monocytes.

Supplementary Figure 4: The brain and ileum share a cluster of migrating macrophages.

Supplementary Figure 5: CD11c cells migrate from the brain to the ileum.

Supplementary Figure 1

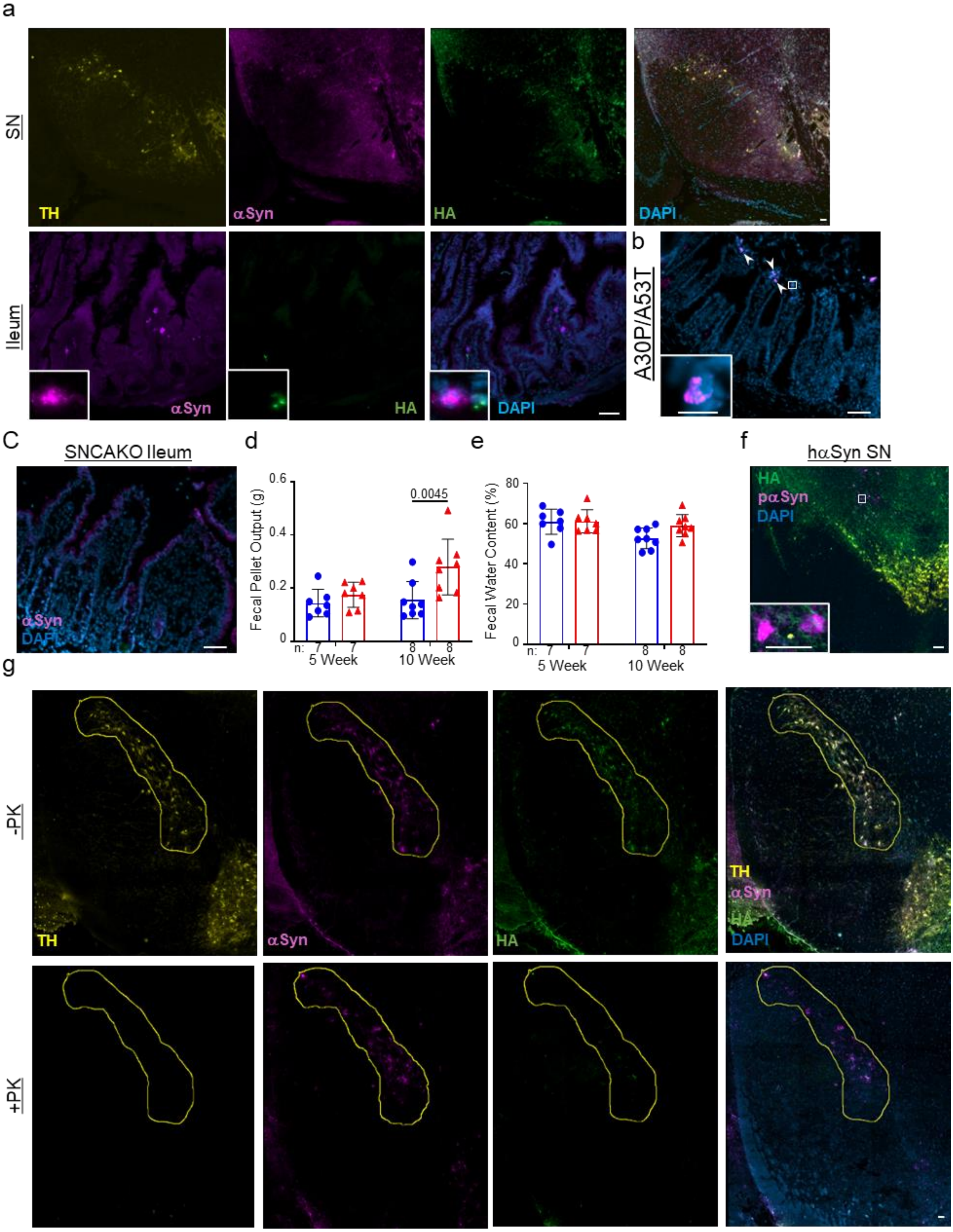

Supplementary Figure 1: αSyn aggregation is specific to the ileum. a) Split channel images for the 5 week hαSyn SN and ileum immunofluorescence images shown in Fig. 1A. b) Representative immunofluorescence for αSyn (magenta) and DAPI (blue) in the ileum of an A30P/A53T heterozygous mouse (b) or a SNCAKO mouse (c) (n=3 mice). d) Fecal pellet output and fecal water content (e) from EV (blue) or hαSyn mice (red) at 5 or 10 weeks after injection. f) Representative

immunofluorescence of pαSyn (magenta), HA (green), and DAPI (blue) in the SN of hαSyn mice, 10 weeks following OP (n=4 mice). g) Split channel images for the PK-resistant ileum images in Figure 2A, with the pars compacta outlined in yellow. Statistical analysis by two-way ANOVA with Bonferroni's post-hoc test. Scale bars represent 50µm in the large images and 20µm (d) or 10µm (a) in the insert. Data are presented as mean values +/- SEM. Source data are provided as a Source Data file.

Supplementary Figure 2

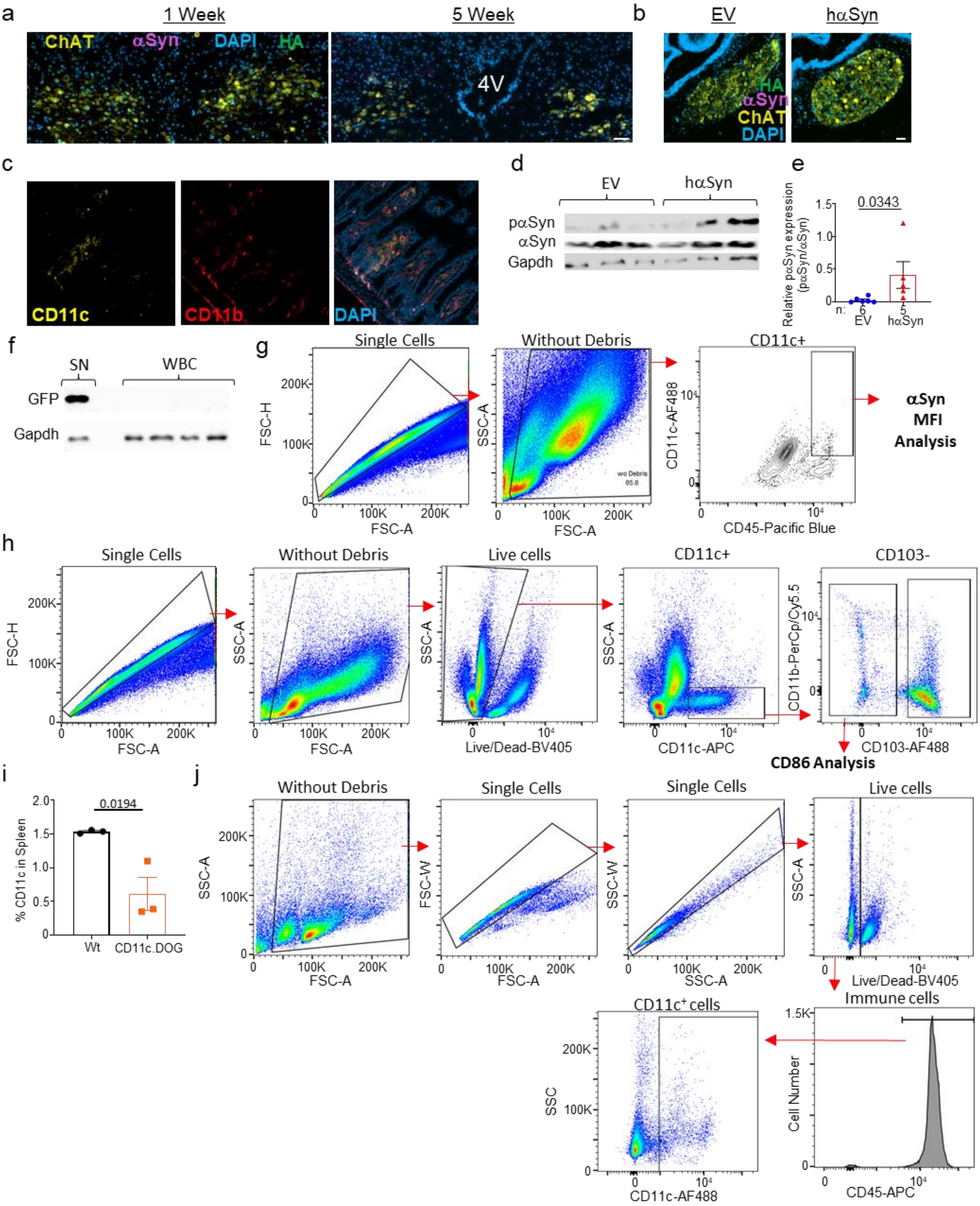

Supplementary Figure 2: αSyn localizes to the region of the ileum enriched with immune cells. a) Representative image of immunofluorescence staining for ChAT (yellow), αSyn (magenta), and HA (green) in the dorsal motor nucleus of the vagus nerve of hαSyn mice, 1 week and 5 weeks after AAV injection. The fourth ventricle is indicated by "4V" (n=4 mice). b) Representative image of immunofluorescence staining for ChAT (yellow), αSyn (magenta), and HA (green) in the vagus nerve of EV or hαSyn animals 5 weeks post-injection (n=3 mice). c) Representative images of CD11b and CD11c in addition to DAPI in the distal ileum of WT animals (n=3 mice). d) representative western blot pictures and analysis (e) from either EV or hαSyn WBC samples for αSyn, pαSyn, and GAPDH (n=3 samples). f) Western Blot analysis of GFP and GAPDH from the SN (first lane) and the WBCs from GFP animals (n=4 samples). g) FACs gating strategy for the aSyn analysis in Fig. 3b & 3c. h) FACs gating strategy for the CD11c^{+} macrophages analyzed in Fig. 3f. i) Quantification of CD11c+ cells in the spleen of CD11c.DOG mice j) FACs gating strategy for the sorted cells used for the scRNA-Seq. Scale bars represent 50µm. Statistical analysis by one-way student's t-Test (e, i). Data are presented as mean values +/- SEM. Source data are provided as a Source Data file.

Supplementary Figure 3

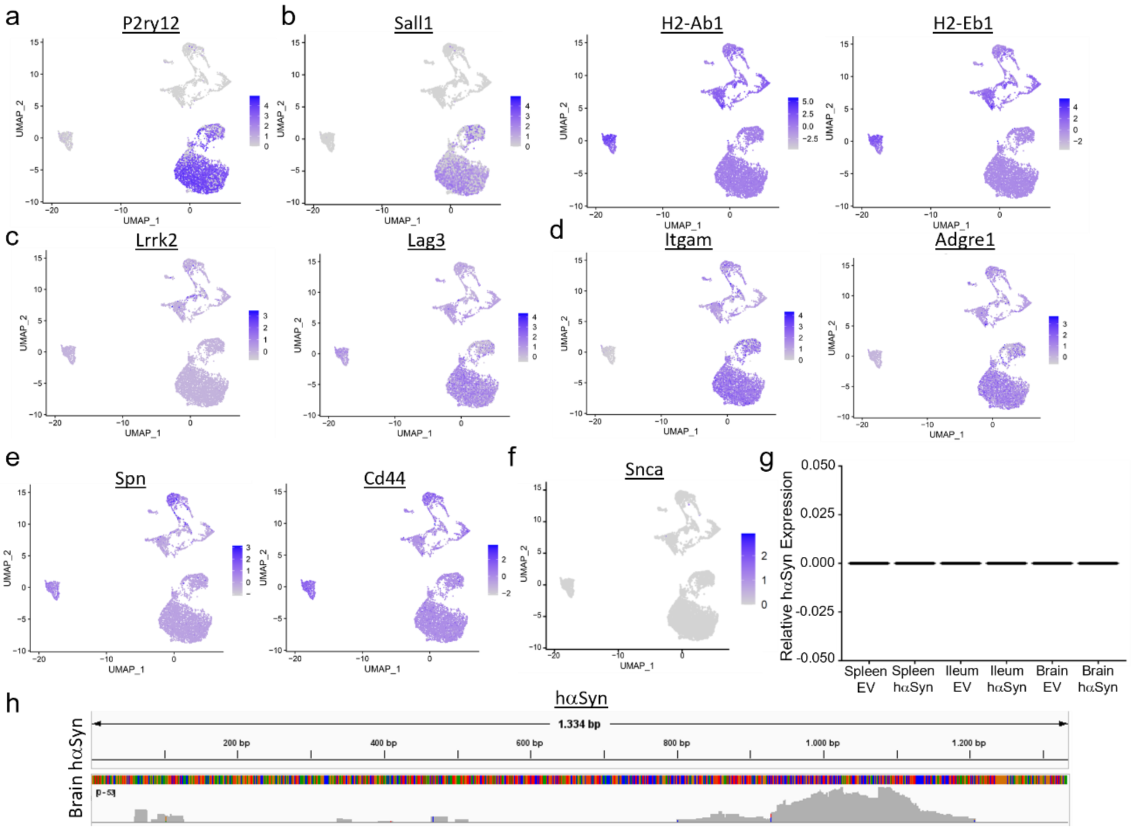

Supplementary Figure 3: CD11c⁺ cells express classical markers for macrophages, dendritic cells, and monocytes. a) UMAP plot for a microglia maker (a), dendritic cells markers (b), PD-associated genes (c), macrophage markers (d), and monocyte markers (e). f) UMAP plot of the expression of the gene encoding αSyn, *Snca*. g) Violin Plot of the hαSyn expression level in all samples. h) Depiction of the reads mapped to the complete hαSyn gene in the Brain hαSyn CD11c samples. In UMAP plots, genes are color-coded by expression, with grey representing not expressed and purple representing expressed.

Supplementary Figure 4

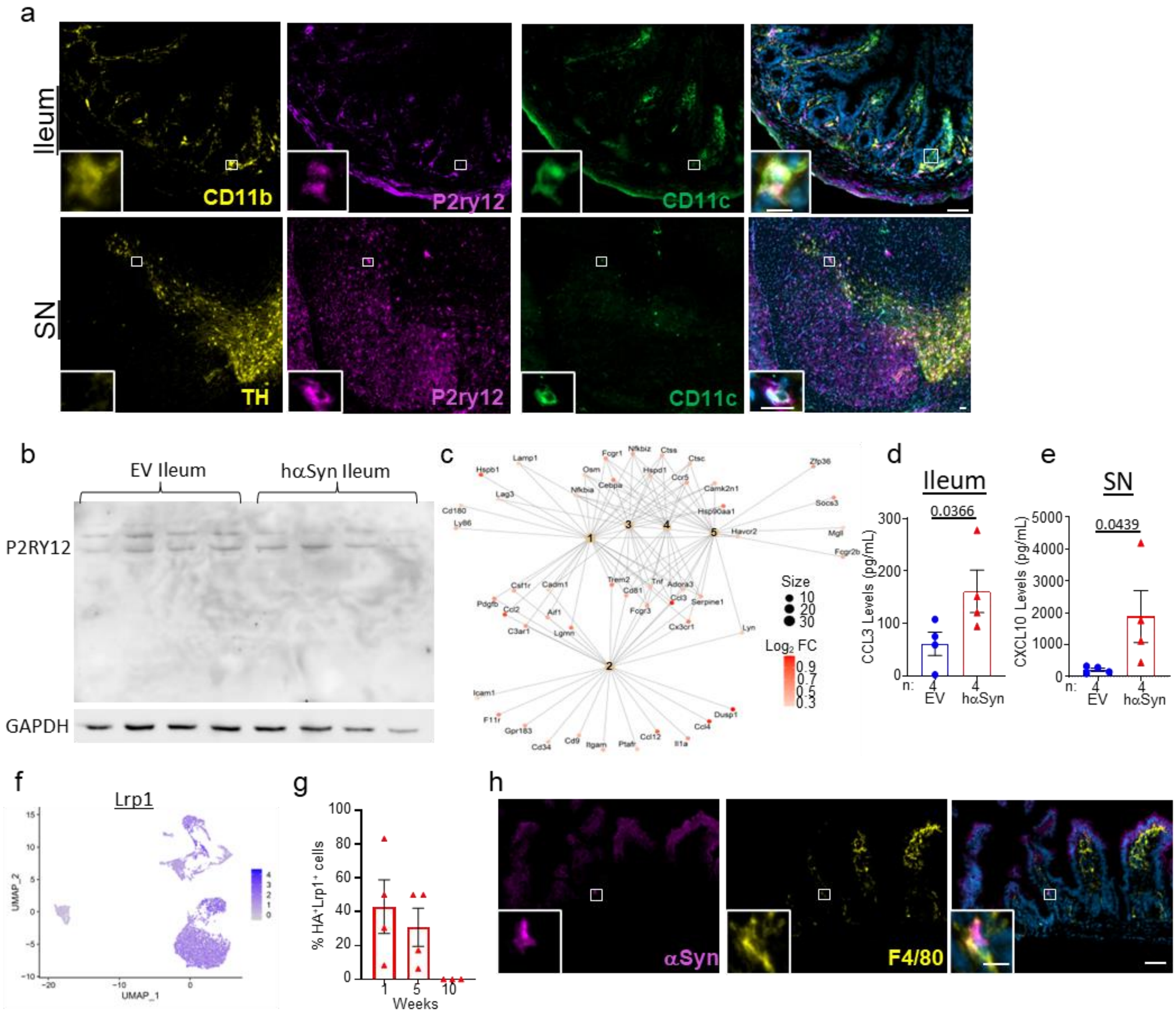

Supplementary Figure 4: The brain and ileum share a cluster of migrating macrophages. a) Representative IHC images from the ileum (top) and SN (bottom) stained for CD11b or TH (yellow), P2RY12 (magenta), CD11c (green). b) Western Blot analysis for P2RY12 (top) and GAPDH (bottom) in protein samples isolated from either EV or hαSyn animals 5 weeks after injection (n=4/group). c) Network plot for top 5 upregulated GO terms enriched in TRM 1 (Fig. 4e). Shading represents Log2 FC, while size of the GO Term dots represents # of genes within the term. d) Quantification of the CCL3 levels in EV and hαSyn Ileal samples. e) Quantification of the CXCL10 levels in EV and hαSyn SN samples. f) UMAP Plot for *Lrp1*. g) Quantification of the percent of HA^+ cells that are also $LRP1^+$ in hαSyn animals 1, 5, and 10 weeks after injection (n=4 for weeks 1 and 5; n=3 for week 10). h) Representative IHC images of F4/80 (yellow) and αSyn (magenta) in hαSyn animals (n=3 mice). Large dots represent GO terms as numbered in Fig. 4e and small dots are genes colored by expression. Scale bars represent 50µm in the large images and 10µm in the zoomed in image. Data are presented as mean values +/- SEM. Statistical analysis by one-way ANOVA with Tukey's post-hoc (g) and one-way student's t-Test (d,e). Source data are provided as a Source Data file.

Supplementary Figure 5

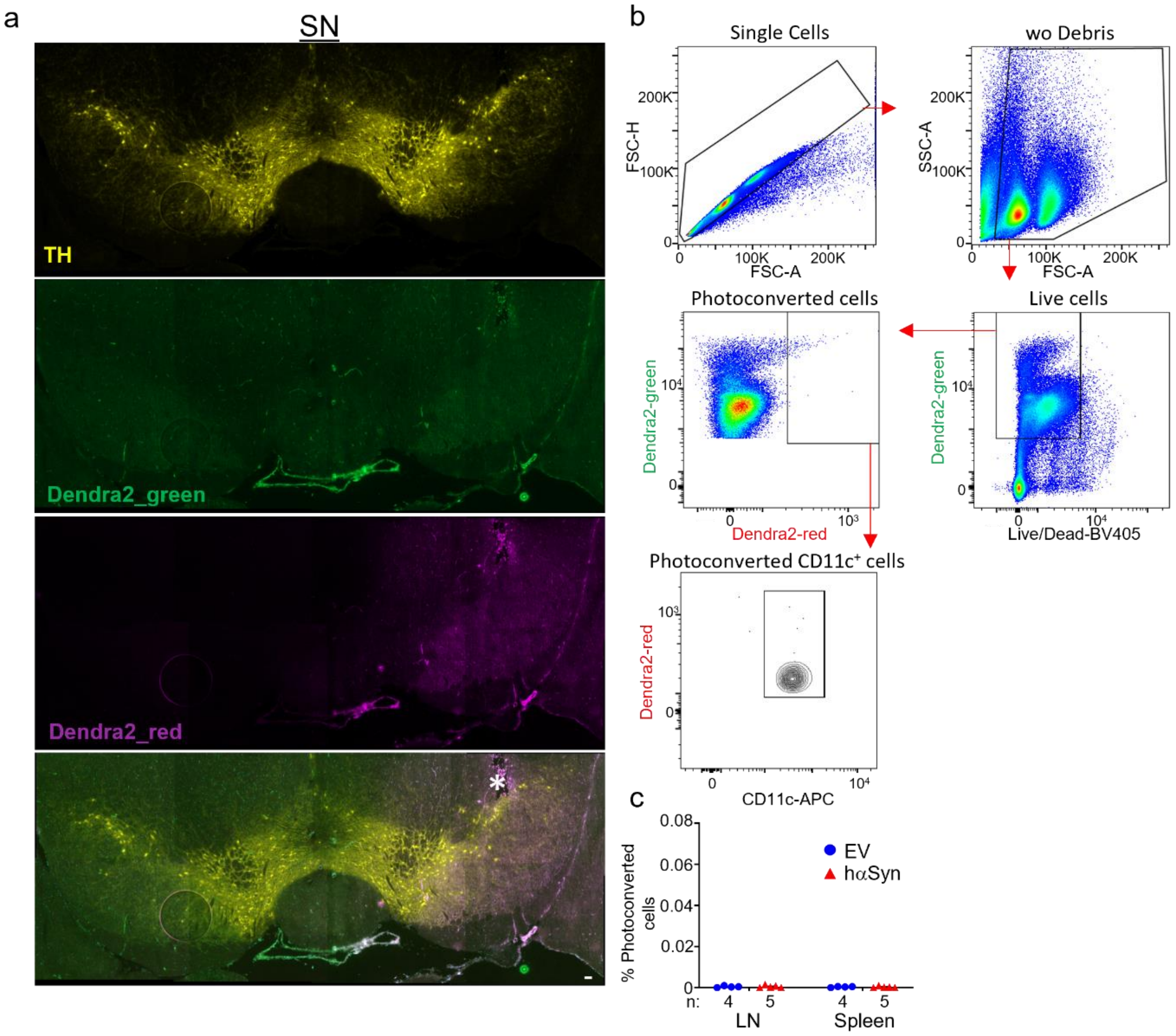

Supplementary Figure 5: CD11c cells migrate from the brain to the ileum. a) Representative images of TH (yellow), Dendra2_green (green), and Dendra2_red (magenta) in the SN of an Dendra2 animal following four weeks of photoconversion (n=3). * indicates optic fiber placement. b) FACs gating strategy for photoconverted Dendra2 in CD11c cells. c) Quantification of photoconverted CD11c cells in the LN and spleen of AV (blue) and hasyn (red) animals. Scale bar represents 50μm. Data are presented as mean values +/- SEM. Statistical analysis by two-way ANOVA with Bonferroni's post-hoc test (c). Source data are provided as a Source Data file.
