# Supplementary material for: Brain-to-gut trafficking of alpha-synuclein by CD11c+ cells in a mouse model of Parkinson’s disease
Source: Nat Commun. 2023 Nov 20;14:7529. doi: 10.1038/s41467-023-43224-z (PMC10658151; doi:10.1038/s41467-023-43224-z)
Supplement: Supplementary file 3 — Description of Additional Supplementary Files [file 41467_2023_43224_MOESM3_ESM.pdf]

Description of Additional Supplementary Files

Supplementary Data 1: Top20markersmono tab depicts the top 20 gene markers for each cluster (clusters are ordered in the same way as in Fig. 4a). GOMono0 tab depicts the top GO terms for the TRM 1 cluster, GOMono3 depicts the top GO terms for the Macrophage 1 cluster. Top10GOTerms depicts the top 10 GO Terms from each of the $CD11c^+$ cell clusters. Enrichment was determined using a one-sided hypergeometric test and the Benjamini-Hochberg correction for multiple comparisons.
