# Supplementary material for: Brain-to-gut trafficking of alpha-synuclein by CD11c+ cells in a mouse model of Parkinson’s disease
Source: Nat Commun. 2023 Nov 20;14:7529. doi: 10.1038/s41467-023-43224-z (PMC10658151; doi:10.1038/s41467-023-43224-z)
Supplement: Supplementary file 5 — Reporting Summary [file 41467_2023_43224_MOESM5_ESM.pdf]

nature portfolio

Corresponding author(s): Chi Wang Ip

Last updated by author(s): Oct 25, 2023

Reporting Summary

Nature Portfolio wishes to improve the reproducibility of the work that we publish. This form provides structure for consistency and transparency in reporting. For further information on Nature Portfolio policies, see our Editorial Policies and the Editorial Policy Checklist.

Statistics

For all statistical analyses, confirm that the following items are present in the figure legend, table legend, main text, or Methods section.

n/a	Confirmed	
☐	☒	The exact sample size (*n*) for each experimental group/condition, given as a discrete number and unit of measurement
☐	☒	A statement on whether measurements were taken from distinct samples or whether the same sample was measured repeatedly
☐	☒	The statistical test(s) used AND whether they are one- or two-sided<br>*Only common tests should be described solely by name; describe more complex techniques in the Methods section.*
☒	☐	A description of all covariates tested
☐	☒	A description of any assumptions or corrections, such as tests of normality and adjustment for multiple comparisons
☐	☒	A full description of the statistical parameters including central tendency (e.g. means) or other basic estimates (e.g. regression coefficient) AND variation (e.g. standard deviation) or associated estimates of uncertainty (e.g. confidence intervals)
☐	☒	For null hypothesis testing, the test statistic (e.g. *F*, *t*, *r*) with confidence intervals, effect sizes, degrees of freedom and *P* value noted<br>*Give P values as exact values whenever suitable.*
☒	☐	For Bayesian analysis, information on the choice of priors and Markov chain Monte Carlo settings
☒	☐	For hierarchical and complex designs, identification of the appropriate level for tests and full reporting of outcomes
☐	☒	Estimates of effect sizes (e.g. Cohen's *d*, Pearson's *r*), indicating how they were calculated

Our web collection on statistics for biologists contains articles on many of the points above.

Software and code

Policy information about availability of computer code

Data collection	No software was used to collect the data in this study.
Data analysis	Flow cytometry analysis was performed using Flowjo v. 10.8.1 (BD Biosciences). Sequencing data were demultiplexed using the CellRanger software v. 6.1.2 and analyzed using Seurat v. 4.1.1 R package and cluster profiler v. 4.8.3. Cytokine analysis by IsoLight software version 1.10.0. Graphpad 9.4.0 was utilized to make all graphs and for statistical analysis. Imaga analysis via Fiji (ImageJ v. 2.3.0).

For manuscripts utilizing custom algorithms or software that are central to the research but not yet described in published literature, software must be made available to editors and reviewers. We strongly encourage code deposition in a community repository (e.g. GitHub). See the Nature Portfolio guidelines for submitting code & software for further information.

Data

Policy information about availability of data

All manuscripts must include a data availability statement. This statement should provide the following information, where applicable:

- Accession codes, unique identifiers, or web links for publicly available datasets
- A description of any restrictions on data availability
- For clinical datasets or third party data, please ensure that the statement adheres to our policy

The sequencing datasets generated and analyzed during the current study are available at GEO under the accession number GSE232840 (https://www.ncbi.nlm.nih.gov/geo/query/acc.cgi?acc=GSE232840). The mm10 Genome used is publicly available (https://www.ncbi.nlm.nih.gov/datasets/genome/GCF_000001635.20/). The full haSyn sequence is available in Source Data. Other source data are provided with this paper. The experiment data that support the

findings of this study are available upon request.

Research involving human participants, their data, or biological material

Policy information about studies with human participants or human data. See also policy information about sex, gender (identity/presentation), and sexual orientation and race, ethnicity and racism.

Reporting on sex and gender	Information of the sex of the individuals from which the autopsies were obtained were available and is present in the methods section. Disaggregated data are provided in the source data file. The self-reported male/female ratio in the control group was 3/1, in the PD group 7/3.
Reporting on race, ethnicity, or other socially relevant groupings	This information was not relevant for our analysis of the biopsies.
Population characteristics	The mean (± standard deviation) age was 74.3 ± 7.7 years in the control group and 73.0 ± 12.4 years in the PD group.
Recruitment	Autopsies were collected from the Department of Neuropathology Marburg.
Ethics oversight	Human brains were obtained with the written informed consent by the reposective responsible dependent and according to the guidelines of the Local Ethics Committee of the University Hospital of Marburg

Note that full information on the approval of the study protocol must also be provided in the manuscript.

Field-specific reporting

Please select the one below that is the best fit for your research. If you are not sure, read the appropriate sections before making your selection.

- [x] Life sciences
- [ ] Behavioural & social sciences
- [ ] Ecological, evolutionary & environmental sciences

For a reference copy of the document with all sections, see nature.com/documents/nr-reporting-summary-flat.pdf

Life sciences study design

All studies must disclose on these points even when the disclosure is negative.

Sample size	Sample sizes were determined from prospective power calculations based on data from comparable experiments.
Data exclusions	The presence of outliers was tested using the GROUT method in Graphpad. Flow cytometry sampled that did not reach the minimum amount of cells for the target population were excluded.
Replication	Replicates in graphs represent independent animals/human samples. For animal experiments, replicates were performed on at least two different days to ensure reproducibility. For the PD ileum patient data in Figure 3, this is representative of only one patient due to the lack of PD ileal samples available to us. The single cell sequencing data is a pool of cells from five biological replicates/ condition.
Randomization	Animals were randomly assigned to various groups. Patient autopsies were assigned to their respective groups based on neuropathological characterization of Parkinson's Disease or not (Control). Parkinson's Disease autopsies were consecutively collected. Control autopsies were selected to match for covariants such as age, sex, and interval from death to tissue fixation.
Blinding	For the B6a.CD11c.DOG mice the scorer was blinded to genotype throughout the experiment (for data collection and analysis). For all other assessment in the animal investigators were blinded to experimental group during the data analysis by assigning the animals to unique numbers. The neuropathologist assessing the CD11c+aSyn+ cells in the patient brain autopsies was also blinded.

Reporting for specific materials, systems and methods

We require information from authors about some types of materials, experimental systems and methods used in many studies. Here, indicate whether each material, system or method listed is relevant to your study. If you are not sure if a list item applies to your research, read the appropriate section before selecting a response.

Materials & experimental systems

n/a	Involved in the study
	☒ Antibodies
☒	☐ Eukaryotic cell lines
☒	☐ Palaeontology and archaeology
	☒ Animals and other organisms
☒	☐ Clinical data
☒	☐ Dual use research of concern
☒	☐ Plants

Methods

n/a	Involved in the study
☒	☐ ChIP-seq
	☒ Flow cytometry
☒	☐ MRI-based neuroimaging

Antibodies

Antibodies used

chicken anti-TH (1:500, Abcam #ab76442), rabbit anti-Syn (1:10,000 brain and 1:5000 other organs, Sigma #S3062), rabbit anti-GFP (1:500, Invitrogen #A11122), rat anti-HA (1:100, Roche, #11867423001), rabbit anti-phospho aSyn S129 (1:100, Abcam, #ab59264), hamster anti-CD11c Alexa Fluor 488 (1:300, Invitrogen, #53-0114-82), rat anti-CD11b (1:100, Serotec, #MCA74G), goat anti-CHAT (1:300, Millipore, #Ab144P), rabbit anti-P2RY12 (1:200, Invitrogen, #PA5-77671), rabbit anti-LRP1 (1:100, Abcam, #Ab92544), rat ant-CD8 (1:500, Serotec, #MCA609G), rat anti-F4/80 (1:300, Serotec, #MCAP497), anti-CD4 (1:1000, Serotec, MCA1767), goat anti-rabbit Cy3 (1:300, Abcam, #ab150175), goat anti-chicken AF647 (1:300, Abcam #ab150175), goat anti-rat AF647 (1:300, Abcam, #ab15016), donkey anti-goat (1:300, Dianova, #705-165-147), rabbit anti-CD11c (1:1000; Abcam EP1347Y; ab52632), mouse alpha-synuclein (1:5000; Roboscreen; Mab 5G4), rabbit anti-phospho aSyn S129 (1:1000, Abcam, #ab59264), rabbit anti-aSyn antibody (1:200, Abcam #212184), rabbit anti-P2RY12 (1:500, Invitrogen, #PA5-77671), rabbit anti-GFP (1:500, Invitrogen #A11122), mouse anti-GAPDH (1:15000, Calbiochem, #CB1001), anti-rat IgG (1:5000, Jackson Immuno research, #712-035-150), anti-rabbit IgG (1:5000, Jackson Immuno Research, #711-035-152), anti-mouse IgG (1:5000, Jackson Immuno Research, 715-035-150), CD16/32 (1:200, Biolegend #101302), Fixable Violet Dead Cells Stain kit (1:1000, Invitrogen, L34955), rat anti-I-A/I-E Cy7(1:100, Biolegend, 107630), rat anti-CD86 PE (1:50, Invitrogen, 12-0862-82), hamster anti-CD103 AF488 (1:100, Biolegend, 121408), hamster anti-CD11b PerCP/Cy55.5 (1:50, Biolegend, #101228), hamster anti-CD11c APC (1:100, Biolegend, 117310), rat anti-CD45 APC (1:200, Biolegend #103111), rat anti-CD4 PE (1:100, BD Biosciences #553049), rat anti-CD8 PerCp/Cy5.5 (1:100, Biolegend #100733) monoclonal rabbit anti-aSyn antibody (1:200, Abcam #212184) , goat anti-rabbit Cy5 antibody (1:300, Jackson Biozol #711-175-152).

Validation

All antibodies used were commercially availbale and previously validated for the species/specificty (see citations provided on manufactorers website). Appropriate positive and negative controls were performed on each tissue type analyzed. Controls utilizing only primary or only secondary antibodies were performed to test for false positive signal. For aSyn, SNCAKO tissue was utilized to verify specificity (Supplementary Figure 1C). The HA specificity was validated using tissue from control animals that did not express HA (Figure 1A &D).

Animals and other research organisms

Policy information about studies involving animals; ARRIVE guidelines recommended for reporting animal research, and Sex and Gender in Research

Laboratory animals

Ten to twelve week old male C57Bl/6J or SNCAKO (JAX stock#016123)45 mice were obtained from Charles River Laboratories (Sulzfeld, Germany). For the transgenic A30P/A53T PD mouse model, male and female heterozygous hm2a-Syn-39 were obtained from Jackson Laboratory (Jax stock# 008239) and 16-17 month old animals used for the experiments. Homozygous male C57Bl/6 mito-Dendra2 mice were obtained from Jackson Laboratories (Jax stock# 018397)47. C57Bl/6J CD11c.DOG mice were kindly provided by G. Hämmerling, interbred with albino C57Bl/6J mice and maintained in the Center for Experimental Molecular Medicine (ZEMM) animal facility at Würzburg University. Both the mito-Dendra2 and the CD11c.DOG mice were male 10-12 weeks old at the time of stereotactic injection. Mice were kept at the animal facility of the Centre for Experimental Molecular Medicine, University of Würzburg, under barrier conditions and at a constant cycle of 12h in the light and 12h in the dark. Colonies were maintained at 20-24 degrees Celsius and 40-60% humidity, with free access to food and water.

Wild animals

No wild animals were used in these studies.

Reporting on sex

Only male mice were used in the experiments described in this manuscript. Except for the A30P/A53T animals where both males and females were used.

Field-collected samples

No field-collected samples were used in this study.

Ethics oversight

The local authorities at the Regierung von Unterfranken, Würzburg, Germany approved all animal experiments.

Note that full information on the approval of the study protocol must also be provided in the manuscript.

Flow Cytometry

Plots

Confirm that:

- [x] The axis labels state the marker and fluorochrome used (e.g. CD4-FITC).
- [x] The axis scales are clearly visible. Include numbers along axes only for bottom left plot of group (a 'group' is an analysis of identical markers).
- [x] All plots are contour plots with outliers or pseudocolor plots.
- [x] A numerical value for number of cells or percentage (with statistics) is provided.

Methodology

Sample preparation	Ten-centimeter-long sections of the ileum were isolated as described above and placed on a PBS soaked paper towel. Feces, fat, and peyers patches were removed and ilea were placed in ice cold RPMI (Sigma #R8758) supplemented with 10% Fetal Bovine Serum (Sigma #F7524) (complete RPMI). The epithelial layer was removed by incubating the tissues at 37°C for 30min in complete RPMI supplemented with 2mM EDTA (Millipore #324506). Ilea were then washed three times with PBS and incubated for 15min at 37°C in Accutase (Sigma #A6964). Digested samples were then passed over a 70um cell strainer, centrifuged, and resuspended in 40% Percoll (GE Healthcare #17-0891-02). Samples were centrifuged at 800 G for 25 min at RT and the pellet containing the leukocytes was collected for further experiments.<br>Brains were isolated as described above and placed in ice-cold PBS. After finely mincing the brains, Accutase was added and the brains were incubated at 37°C for 30min while shaking. Following digestion, brains were pressed through a 70um cell strainer and resuspended in 40% Percoll. Samples were centrifuged at 650G for 25 minutes and the pellets were isolated and used for further experiments.<br>Spleens and LN were pressed through a 70um filter and incubated with red blood cell (RBC) lysis buffer (0.15M NH4Cl, 10mM KHCO3, 0.1mM Na2EDTA) for 3 minutes. Cells were centrifuged down and used for further experiments.<br>Peripheral blood was incubated in RBC lysis buffer for 5 min prior to centrifugation.
Instrument	BD FACSLyric
Software	Flowjo v. 10.8.1 (BD Biosciences)
Cell population abundance	Approximately 20,000 CD11c cells were sorted from the various samples. Purity of the samples was determined based on transcriptomic profiles and contaminating cell populations were filtered out.
Gating strategy	All gating strategies are provided in the supplemental figures. The positive and negative gatings were set using fluorescence minus one controls.

- [x] Tick this box to confirm that a figure exemplifying the gating strategy is provided in the Supplementary Information.
